# A Novel Stress-Diathesis Model to Predict Risk of Post-operative Delirium: Implications for Intra-operative Management

**DOI:** 10.3389/fnagi.2017.00274

**Published:** 2017-08-18

**Authors:** Renée El-Gabalawy, Ronak Patel, Kayla Kilborn, Caitlin Blaney, Christopher Hoban, Lawrence Ryner, Duane Funk, Regina Legaspi, Joseph A. Fisher, James Duffin, David J. Mikulis, W. Alan C. Mutch

**Affiliations:** ^1^Department of Anesthesia and Perioperative Medicine, Max Rady College of Medicine, University of Manitoba Winnipeg, MB, Canada; ^2^Department of Clinical Health Psychology, Rady Faculty of Health Sciences, University of Manitoba Winnipeg, MB, Canada; ^3^Department of Physics and Astronomy, Faculty of Science, University of Manitoba Winnipeg, MB, Canada; ^4^Department of Anesthesia, Faculty of Medicine, University of Toronto Toronto, ON, Canada; ^5^Department of Physiology, Faculty of Medicine, University of Toronto Toronto, ON, Canada; ^6^Department of Medical Imaging, Faculty of Medicine, University of Toronto Toronto, ON, Canada; ^7^Canada North Concussion Network Winnipeg, MB, Canada

**Keywords:** post-operative delirium, neuropathophysiology, peri-operative care, neuropsychological, stress-diathesis, neuroimaging

## Abstract

**Introduction:** Risk assessment for post-operative delirium (POD) is poorly developed. Improved metrics could greatly facilitate peri-operative care as costs associated with POD are staggering. In this preliminary study, we develop a novel stress-diathesis model based on comprehensive pre-operative psychiatric and neuropsychological testing, a blood oxygenation level-dependent (BOLD) magnetic resonance imaging (MRI) carbon dioxide (CO_2_) stress test, and high fidelity measures of intra-operative parameters that may interact facilitating POD.

**Methods:** The study was approved by the ethics board at the University of Manitoba and registered at clinicaltrials.gov as NCT02126215. Twelve patients were studied. Pre-operative psychiatric symptom measures and neuropsychological testing preceded MRI featuring a BOLD MRI CO_2_ stress test whereby BOLD scans were conducted while exposing participants to a rigorously controlled CO_2_ stimulus. During surgery the patient had hemodynamics and end-tidal gases downloaded at 0.5 hz. Post-operatively, the presence of POD and POD severity was comprehensively assessed using the Confusion Assessment Measure –Severity (CAM-S) scoring instrument on days 0 (surgery) through post-operative day 5, and patients were followed up at least 1 month post-operatively.

**Results:** Six of 12 patients had no evidence of POD (non-POD). Three patients had POD and 3 had clinically significant confusional states (referred as subthreshold POD; ST-POD) (score ≥ 5/19 on the CAM-S). Average severity for delirium was 1.3 in the non-POD group, 3.2 in ST-POD, and 6.1 in POD (F-statistic = 15.4, *p* < 0.001). Depressive symptoms, and cognitive measures of semantic fluency and executive functioning/processing speed were significantly associated with POD. Second level analysis revealed an increased inverse BOLD responsiveness to CO_2_ pre-operatively in ST-POD and marked increase in the POD groups when compared to the non-POD group. An association was also noted for the patient population to manifest leucoaraiosis as assessed with advanced neuroimaging techniques. Results provide preliminary support for the interacting of diatheses (vulnerabilities) and intra-operative stressors on the POD phenotype.

**Conclusions:** The stress-diathesis model has the potential to aid in risk assessment for POD. Based on these initial findings, we make some recommendations for intra-operative management for patients at risk of POD.

## Introduction

Post-operative delirium (POD) remains a poorly understood and highly variable neuropsychiatric syndrome characterized by neurocognitive dysfunction, which can include fluctuating disturbances in attention, awareness, thinking, and psychomotor behavior that frequently manifests in the hours or days following surgery. Proper classification of the condition remains problematic and may encompass a spectrum of acute confusional states and low-grade encephalopathic states (Martins and Fernandes, 2012). This has been referred to as subsyndromal or subthreshold POD in prior research and more recently described as Attenuated Delirium Syndrome in the Diagnostic and Statistical Manual of Mental Disorder 5th Edition (DSM-5; American Psychiatric Association, 2013). Indeed, recent research assessing a spectrum of severity of POD suggests that poor outcomes are associated with confusional states not meeting full POD criteria (Inouye et al., 2014a), emphasizing the importance of a graded severity assessment. Risk prediction, prevention and optimal peri-operative course to minimize these related problems are largely unknown. The individual and societal costs of management of these problems are substantial (Young and Inouye, 2007; Dasgupta and Hillier, 2010) and the health care costs soar into the billions of dollars annually (Leslie et al., 2008). POD has been conceptualized as a “disease of the elderly” because of its high prevalence in late life and that older adults with dementing disorders and/or vascular compromise are particularly prone to the risks of POD (Beals et al., 2003; Olney et al., 2004; Baranov et al., 2009; Lei et al., 2014; Strøm et al., 2014; Fong et al., 2015). Historically, the expression of POD has hinged on the concept that anesthetic agents may be toxic to brain tissue. This study proposes a novel conceptualization of POD that moves away from the contribution of the anesthetic agents themselves to critically examine the conduct of anesthesia, and how this management may interact with existing risks, to impact the vulnerable brain. We provide preliminary data to support this novel hypothesis.

Recently big data retrospective clinical studies and meta-analyses would suggest that neurotoxicity related to anesthetic exposure does not predict POD (Mason et al., 2010). Prior investigations have been largely driven by preclinical animal models, and neuronal dropout indicative of anesthetic neurotoxicity may not translate to POD in humans (Jevtovic-Todorovic, 2016). Further, in recent years, clinical studies investigating neuroprotection of anesthetic agents have also been criticized because of the questionable quality of these trials (Ishida et al., 2014). Prospective studies to examine anesthetic depth or the potential protective effects of chosen agents such as ketamine have resulted in null findings (e.g., Avidan et al., 2017) and findings are mixed in the research that does exist. There is an evident need to investigate other potential mechanisms.

Our partial understanding of POD is partly attributable to the fact that research to date has been limited in its comprehensiveness. First, there has been only partial use of major advancements in neuroimaging techniques. A recent review of the literature published in the *Lancet* (2014) stresses the importance of these advanced neuroimaging techniques for shedding light on the pathophysiological complexities that seem to exist for POD (Inouye et al., 2014b). Preliminary evidence from case series have suggested that cerebral blood flow may play an important role in POD (Yokota et al., 2003; Fong et al., 2006). In one recent retrospective study, abnormalities in cerebral blood flow were identified as contributing to neurological complications post-operatively including POD (Xu et al., 2015), yet this has not been in a sophisticated manner. As previously indicated, another shortcoming of prior research relates to a limited scope in POD nosology as a discrete entity. The National Institute of Mental Health initiated the Research Domain Criteria to urge researchers to conceptualize mental disorders more broadly outside the confines of the DSM with a focus on symptomatology, model mental illnesses as brain disorders and identify syndromes based on pathophysiological findings (Insel et al., 2010). This emphasizes the importance of broadening current understanding of POD and examining POD on a spectrum of severity to include acute confusional states and low-grade encephalopathic states.

In this preliminary study we document our comprehensive approach to establish a stress-diathesis (vulnerability) multifactorial model predictive of POD in patients having major surgery. The concept of a stress-diathesis model has been used widely in psychological research accounting for the dynamic interplay between pre-existing dispositions or vulnerabilities and the role of environmental stressors acting as catalysts in the expression of a particular condition. A large body of research has established critical pre-operative risk factors in adults such as pre-operative cognitive dysfunctions (Dasgupta and Dumbrell, 2006; Inouye et al., 2014b; Jones et al., 2016), history of psychiatric illness, and illicit drug use (Inouye et al., 2014b; O'Sullivan et al., 2014). These risk factors have all been shown to be associated with neurodegenerative processes including cerebrovascular dysfunction (Sprooten et al., 2017). The intra-operative period characterizes the stressor in this model, which has also been largely overlooked with respect to a close examination of intra-operative course including changes in critical hemodynamic factors and management of gas exchange during mechanical ventilation, which is hallmark in both surgical and intensive care settings.

This study aims to comprehensively examine critical vulnerability factors established in prior research including all major domains of cognitive functioning through neuropsychological testing and psychiatric history. We also included an examination of the pre-operative brain using blood oxygenation level-dependent (BOLD) magnetic resonance imaging (MRI) while patients underwent regulated CO_2_ exposure (referred to as the “CO_2_ stress test”) to act as a proxy for end-tidal CO_2_ stress during surgery where a paradoxical vasodilatory response may reduce cerebral blood flow affecting vulnerable regions of the brain (referred to as intracranial steal; see Supplemental Data Sheet 1 and Supplemental Figures 1A,B for a description of the approach used in this study). With respect to the proposed stressor, we have monitored intra-operative hemodynamics, end-tidal gas tensions and cerebral oximetry with high fidelity. Finally, we conducted a comprehensive assessment of POD using the Confusion Assessment Method-Severity (CAM-S) to yield continuous scoring to enable identification of both “subthreshold” and full manifestations, and included a follow-up assessment at least 1 month post-operatively. A preliminary predictive model is constructed and recommendations for patient management are advanced.

## Methods

This study was approved by the Biomedical Research Ethics Board (BREB) of the University of Manitoba. This trial is registered at clinicaltrials.gov as NCT02126215.

Patients who were undergoing high-risk surgeries requiring a post-operative stay were approached to participate in the study through the Pre-Anesthesia Clinic (PAC) of the Health Sciences Centre at the Max Rady College of Medicine in Winnipeg, MB. Witnessed informed consent was obtained from each patient. Exclusion criteria included simultaneous planned carotid endarterectomy, carotid stenosis—if previously documented, contraindications to MRI including claustrophobia, and known chronic obstructive lung disease with CO_2_ retention. At the time of consent, patients completed psychiatric symptom measures (described below). Patients were seen pre-operatively in the week prior to surgery for comprehensive neuropsychological testing followed immediately by the MRI BOLD CO_2_ stress test. Patients received a $50 gift card that included coverage for transportation and parking during their pre-operative visit for participation in the study. A trained psychometrist (PhD level clinical psychology graduate student) supervised by a registered Clinical Neuropsychologist administered a battery of neuropsychological tests over the span of approximately 1 hour. Subsequently, the MRI BOLD CO_2_ stress test was initiated. Patients returned for their scheduled surgery. There were no changes to standard surgical procedures; trained research personnel collected intra-operative data. Following surgery, trained research personnel blinded to pre-operative performance on all assessments conducted daily POD assessments for up to 5 post-operative days including day 0 (day of surgery). Patients were subsequently contacted via phone at least 1 month post-operatively and asked about their cognitive functioning since their surgery.

### Diathesis assessments

#### Pre-operative psychiatric and neuropsychological assessments

Upon initial recruitment in PAC, patients completed the validated Patient Health Questionnaire (PHQ-9; Kroenke et al., 2001) to assess depressive symptoms and the Generalized Anxiety Disorder Scale (GAD-7; Spitzer et al., 2006) to assess anxiety symptoms, and self-reported on psychiatric disorder diagnoses previously made by health professionals. They also reported on their history of illicit drug use. Patients were flagged if they were clinically significant on the PHQ-9 and GAD-7 (≥10), reported a psychiatric diagnosis, or who indicated illicit drug use in 2 weeks prior to their surgery.

For neuropsychological testing, attention was assessed using Trails A and Weschler Adult Intelligence Scale (WAIS)-IV Digit Span; information processing speed using WAIS-IV Digit Symbol Coding; verbal memory using Hopkins Verbal Learning Test-Revised; visual construction, planning and organization using Rey's Complex Figure (copy trial); visual memory using Rey's Complex Figure (immediate recall trial); executive functioning/processing speed using Trails B and Delis-Kaplan Executive Function System (DKEFS) Color Word Interference; verbal and semantic fluency using F-A-S and Animal Fluency; spatial skills using CLOX I (free draw) and II (copy); and global cognitive and mental status using the Mini-Mental Status Examination (MMSE). Raw scores were converted to scaled scores and standard scores (dependent on measure assessed). Patients also completed a baseline POD assessment, described below.

#### Pre-operative neuroimaging and CO_2_ stress test

The CO_2_ stress test was conducted during neuroimaging where all participants had model-based prospective end-tidal (MPET) CO_2_ targeting achieved by precise delivery of CO_2_ at a fixed concentration using a sequential breathing circuit regulated by a computerized gas-blender (RespirAct™, Thornhill Research Inc., Toronto, ON) (Slessarev et al., 2007). This device allows precise manipulation of end tidal CO_2_ levels under iso-oxic conditions (see Supplemental Data Sheet 1 for a description of the terms related to gas exchange used in this study)—and a target end tidal O_2_ = 115 mmHg. Monitoring during the imaging period included continuous heart rate and pulse oximetry and non-invasive blood pressure (BP) at 3-min intervals.

All images were acquired using a Siemens Verio 3.0T MR scanner with a 12-channel phased-array head coil. The MRI protocol consisted of baseline anatomical imaging including sagittal 3D T1 magnetization-prepared rapid gradient-echo (MPRAGE) (whole brain coverage; matrix: 256 × 256; slice thickness: 2.2 mm; no interslice gap), axial fluid-attenuated inversion recovery (FLAIR), axial gradient recalled echo planar images (EPI GRE) sequences, and continuous BOLD EPI with MPET. The breathing sequence during BOLD imaging consisted of a triple box-car hypercapnic stimulus (see Supplemental Figure 1A for the sequence used). A video is shown in Supplemental Video 1 demonstrating the dynamic response of the brain regionally to changes in end-tidal CO_2_ tension.

BOLD MRI data was acquired with a T2^*^-weighted single-shot gradient echo pulse sequence with echoplanar (EPI) readout (field of view: 24 × 24 cm; matrix: 64 × 64; TR: 2000 ms; TE: 30 ms; flip angle: 85°; slice thickness: 5.0 mm; interslice gap: 2.0 mm; voxel size 3.75 × 3.75 × 6.0 mm; number of temporal frames = 330). A 30-s lead in for BOLD imaging was undertaken for equilibration and these images were discarded from analysis. The total duration of the MRI assessment was approximately 25 min.

### Stressor assessments

#### Intra-operative assessments

No management constraints were placed on the patient's anesthetic approach. Where appropriate, regional anesthetic supplements were undertaken (nerve blocks or epidurals). All patients received a general anesthetic—either sevoflurane or desflurane as volatile agent in air:O_2_. As per standard of care, no patient was administered N_2_O. All patients received intravenous supplements including propofol and midazolam for induction and muscle relaxants as required. All were endotracheally intubated and mechanically ventilated. All patients had arterial cannulation to record blood pressure continuously, electrocardiography (ECG) monitoring to record heart rate, and infra-red sensors were applied to the forehead bilaterally to measure frontal lobe oxygen saturation (ForeSight monitor). Hemodynamics, end-tidal gas tensions (O_2_, CO_2_ and anesthetic vapor) were recorded at 0.5 hz using a data acquisition system and stored on a laptop computer. The data stream recorded at 0.5 hz included heart rate, systolic, diastolic and mean blood pressure, respiratory rate, tidal volume, end-tidal O_2_ and CO_2_, end-tidal anesthetic vapor, right, left and mean cerebral oxygen saturation. The duration of time in the operating room was also recorded. Concatenated data were examined and we report on mean arterial pressure greater than or less than 60 mmHg, end-tidal CO_2_ delta greater than or less than 5 mmHg, end-tidal vapor concentration greater than or less than one minimum alveolar concentration (MAC)—age adjusted, cerebral saturation greater than or less than 60% and duration of procedure greater than or less than 120 min. As well, hemodynamic and end-tidal data were further examined. The median value for mean blood pressure and end-tidal CO_2_ were assessed. For blood pressure, the pressure below the 10th percentile and above the 90th percentile, and the duration above and below these limits, were determined for each patient as an index of hemodynamic instability. As an index of variability in intra-operative end-tidal CO_2_ control, the duration above or below the median CO_2_ by ± 5 mmHg for the conduct of the intra-operative course was determined for each patient—a reflection of the CO_2_ delta examined by the MRI BOLD CO_2_ stress test employed. The duration of cerebral saturation below 60% O_2_ saturation was also collated. At the end of their surgical procedure all patients were initially monitored in the recovery room and then transferred either to the surgical intensive care, or the inpatient surgical wards. Both intra-operative and total narcotic dose over the course of the hospital stay was calculated for each patient with dosages of the various narcotics used converted to morphine equivalents in mg. The data stream was processed, collated with Excel, and transferred to SPSS for analysis.

### Post-operative assessment for delirium

A trained blinded interviewer conducted the CAM-S, a structured 10-15 min clinical interview, to assess the presence and severity of POD. In cases where the patient was intubated or could not complete the extended CAM-S, the briefer CAM-ICU was administered. With the exception of fluctuation, which is identified as mild (score of 0) or marked (score of 1), all other symptoms are identified as absent (0), mild (1) or marked (2). The total severity score was based on a sum score that could range from 0 through 19. In this study, we report the *peak* post-operative severity score for each patient throughout their inpatient stay and the *average* severity score up to 5 post-operative days (unless discharged prior to 5 days). A diagnosis of full POD was based on the presence of either acute onset of change or symptom fluctuation in mental status, inattention, and either disorganized thinking or altered level of consciousness, in line with prior research. In this sample, full POD was associated with peak severity scores on at least 1 post-operative day that ranged from 8 to 14. Subthreshold delirium (ST-POD) was defined as those not meeting full criteria but displayed elevated severity scores (≥5) on the POD severity long form on at least 1 post-operative day. This clinically significant cutoff has been found to be associated with higher risk of increased length of stay, increased healthcare costs, and post-operative admittance to nursing homes, functional and cognitive decline, and death within 90 days post-operatively compared to lower scores (Inouye et al., 2014a). Patients with ST-POD also met a large proportion of criteria for full POD with the exception of one or two items. For patients who did not want to complete the extended interview, the short severity form was offered and these scores were subsequently weighted on the same metric as the long form. All patients were followed up by phone by a research assistant at least 1 month post-operatively and asked if the patient or a loved one of the patient noticed if there “*was a short or long period of time after you got home that you felt your thinking had changed. For example, this could be changes in your ability to focus your attention and/or being able to keep track of what is being said to you, difficulty staying on one subject while speaking, feeling confused, or problems remembering things. This could even be seeing or hearing things that weren't really there.”* Responses are descriptively reported.

### Statistical analyses

Standard preprocessing of MRI EPI output was accomplished with statistical parametric mapping version 8 (SPM8) software, including batch processing by an SPM toolbox and custom written in-house MatLab scripts. The preprocessing included re-alignment of images, slice time correction, co-registration with the MPRAGE images, re-slicing to the MPRAGE dimensions for both approaches, smoothing and normalization into Montreal Neurological Institute (MNI) space and inclusive masking to assess gray and white matter distribution. Motion artifact was examined. Studies were rejected if motion over the conduct of the study period was greater than 3 mm in any plane. BOLD imaging was processed as 1st and 2nd level analysis by SPM. intra-operative data were concatenated and binned on a minute-by-minute basis.

The structural neuroimaging components from each study were reviewed by a board-certified neuro-radiologist, who indicated a Fazekas score for each patient (see below).

Other data were analyzed using SPSS. Bivariate correlations examined the relationship between cognitive summary scores and psychiatric severity scores as indicated by PHQ-9 and GAD-7 with continuous severity POD measures. If significant results were indicated, we conducted a bivariate linear regression model followed by a model controlling for age, education, sex and pre-operative baseline POD severity. We also conducted analyses of variance to examine mean score differences across primary diathesis and stress factors among those classified as non-POD, ST-POD, and full POD. Finally, to examine the within subjects change from pre-operative CO_2_ delta during the stress test to intra-operative CO_2_ delta by the between-subjects POD groups, we conducted a repeated measures analysis of covariance including age, education and sex as covariates, and additionally: (1) identified cognitive and psychiatric diathesis factors, (2) hypercapnic and inverse hypercapnic voxel responses in gray and white brain matter, and (3) contributory intra-operative factors. Because of restricted sample size, results are shown graphically, and trends are only discussed descriptively.

## Results

### Patient characteristics

Twelve patients completed the protocol. A total of 30 patients undergoing high-risk surgeries were approached in the PAC for enrollment. Thirteen were unwilling to participate, leaving 17 who consented. The majority of patients that were approached and unwilling to participate lived in rural Manitoba, and could not make an additional trip to Winnipeg prior to their surgery. Of the remaining, 5 patients were excluded; 2 had contraindications to the MRI; 1 had surgery canceled; 1 had a scheduling conflict for MRI scan time; and 1 was too nervous to be scanned.

Individual patient characteristics are shown in Table 1 (sociodemographic and diathesis factors for all participants) and Table 2 (intra-operative stress factors for all participants). The mean age of the entire sample was 62 ± 10 years and mean education was 13 years ± 2. There were 7 males and 5 females. The mean duration of surgery was 221 ± 39 min. In 6 of 12 patients the mean BP fell below 60 mmHg on at least one occasion for a minimum of 1 min. In 2 of 12 the cerebral saturation was less than 60% for a minimum of 1 min. The mean CO_2_ delta was 13.2 ± 3.0 mmHg.

**Table 1:** Sociodemographic and diathesis factors among each participant.

				**Neuropsychological testing**		**Voxel counts**								
**Pt**	**Age**	**Sex**	**Psychiatric disorder**	**Semantic fluency (Per)**	**Information processing (Per)**	**Gray + White Matter Type 1**	**Gray + White Matter Type 2**	**Gray matter Type 1**	**Gray Matter Type 2**	**White Matter Type 1**	**White Matter Type 2**	**Total count**	**Fazekas score**	**Delirium**
1	59	F	1	45	37	0	1	0	0	0	1	2	0	POD
2	72	M	0	86.5	43.5	0	0	0	0	0	0	0	0	NON-POD
3	64	M	0	50	63	0	0	0	0	0	0	0	0	NON-POD
4	75	M	0	82	69	1	0	1	0	1	0	3	1	ST-POD
5	60	F	0	50	56.5	1	1	1	1	1	1	6	1	ST-POD
6	56	F	1	55	16	1	0	1	0	1	0	3	0	ST-POD
7	66	M	1	1	1	1	0	1	0	0	1	3	2	POD
8	58	M	0	81	63	0	0	0	0	0	0	0	0	NON-POD
9	77	F	0	84	20	1	0	1	0	1	0	3	2	NON-POD
10	51	F	0	1	N/A	0	0	0	0	0	0	0	0	POD
11	43	M	0	45	50	0	0	0	1	0	0	1	0	NON-POD
12	63	M	0	81	75	0	1	0	0	0	1	2	1	NON-POD

*Pt, patient number; 1, yes; 0, no; Per, percentile; N/A, missing; POD, post-operative delirium; ST, subthreshold. Psychiatric disorder indicated in those with clinically significant levels of depressive or anxiety symptoms, reported diagnosis, or illicit drug use in past 2 weeks. Percentiles are reported for semantic fluency (standard score for Animals) and information processing (DKEFS Color Word Interference mean of standard score on condition 1 and 2). All voxel counts calculated at p = 0.001. Type 1 = response to hypercapnic stimulus (increased BOLD response); Type 2 = inverse response to hyperapnic stimulus (decreased BOLD response). Among voxel counts, 1 = <median for type 1 and >median for type 2 (non-normal); 0 = >median for type 1 and <median for type 2 (normal); total count = number of non-normal voxels*.

**Table 2:** Peri-operative stress factors among each participant.

	**MAP**				**CO**_**2**_**Level**								
**Pt**	**MAP < 60**	**<10th Per**	**>90th Per**	**Total**	**CO_2_ delta**	**5 mmHg less**	**5 mmHg greater**	**Total**	**Mean MAC**	**Sat < 60%**	**Time < 60%**	**Length**	**Delirium**
1	0	12	11	23	9.1	0	4	4	1.1	0	0	107	POD
2	1	36	11	47	17.4	73	14	87	N/A	0	0	376	NON-POD
3	0	8	6	14	13.8	25	33	58	0.9	0	0	78	NON-POD
4	0	45	44	99	20	13	37	50	0.9	0	0	450	ST-POD
5	0	12	11	23	9.8	8	0	8	0.9	0	0	120	ST-POD
6	1	3	6	9	13.7	0	6	6	0.9	0	0	114	ST-POD
7	1	37	38	75	14.5	26	4	30	0.6	1	30	377	POD
8	1	27	27	54	12.4	1	1	2	1.0	0	0	284	NON-POD
9	1	6	5	11	N/A	N/A	N/A	N/A	N/A	0	0	61	NON-POD
10	1	19	18	37	13.2	7	0	7	0.6	1	94	192	POD
11	0	27	13	50	7.5	0	0	0	1.1	0	0	270	NON-POD
12	1	18	19	37	9.2	2	11	13	0.8	0	0	228	NON-POD

Pt, patient; N/A, missing; MAP, mean arterial pressure; Per, percentile; POD, post-operative delirium; MAC, minimum alveolar concentration; ST, subthreshold <10th Per = less than 10th percentile of median MAP for duration of procedure; >90th Per = greater than 90th percentile of median MAP for duration of procedure; 5 mm Hg Less = 5 mm Hg less than the median intra-operative CO_2_ for the duration of the procedure; 5 mm Hg Greater = 5 mm Hg greater than the median intra-operative CO_2_ for the duration of the procedure; Time <60% = time in minutes the cerebral saturation was less than 60% oxygen saturation

### CO_2_ MRI stress test

The end-tidal gas control for each patient and the comparison between the non-POD patients and the combined POD and ST-POD patients is shown in Supplementary Material.

**Figure 1:**
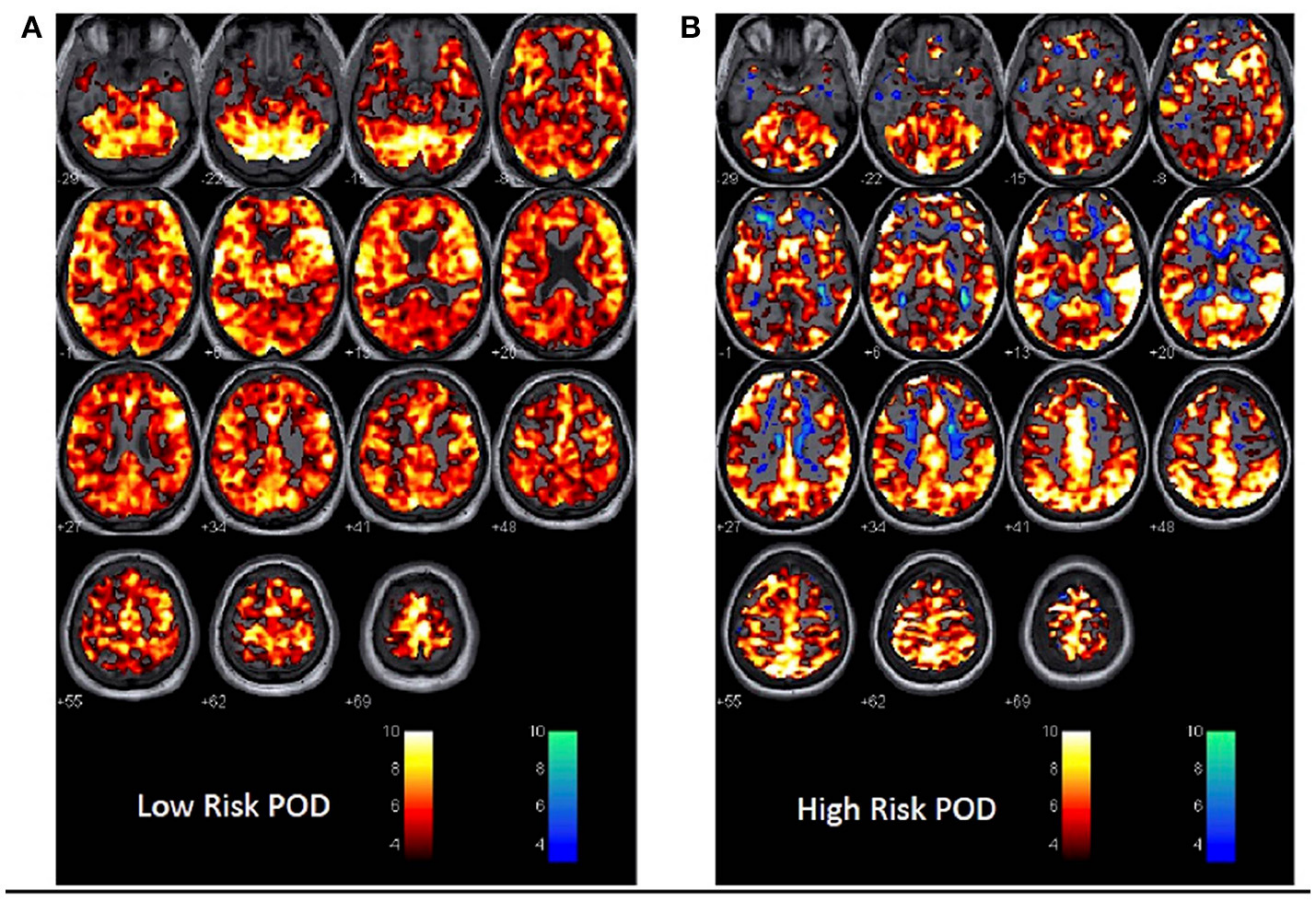
1st Level Analysis in SPM showing response to the CO_2_ stress test in **(A)** a patient at low risk for POD.In this instance the expected response to the CO_2_ stimulus as recorded during BOLD imaging is shown. A vigorous response to CO_2_ is evident from the hot voxel response—shades of orange. The response at the *p* = 0.001 level occurred in 84% of whole brain parenchyma. The numbers below each image are the distance in mm above or below the anterior-posterior commissure. This patient was a non-POD outcome. The color bar is the *t*-value for fit to the general linear model from the SPM analysis. Voxels are colored if the *t*-value exceeded 3.11 in this instance. **(B)** A patient at risk of POD. Here there is less response to the hypercapnic signal—a 64% response to hypercapnia and now an inverse or intracranial steal signal shown in cold voxels—shades of blue. The inverse voxel count was 4.3% of the total count. This patient had a subthreshold POD outcome.

A comparison between a patient deemed low risk and a patient high risk for POD following MRI BOLD CO_2_ stress testing is shown in Figures 1A,B. The expected response to the hypercapnic stimulus at the *p* = 0.001 level is shown in the hot voxels (shades of orange). The inverse response to the hypercapnic stimulus—that is a decrease in BOLD signal with an increase in CO_2_ or vice versa is shown by the cold voxels (shades of blue). See the Supplemental Figures 1A,B for a full description of the approach. The blue voxels are indicative of regions of intracranial steal.

**Figure 2:**
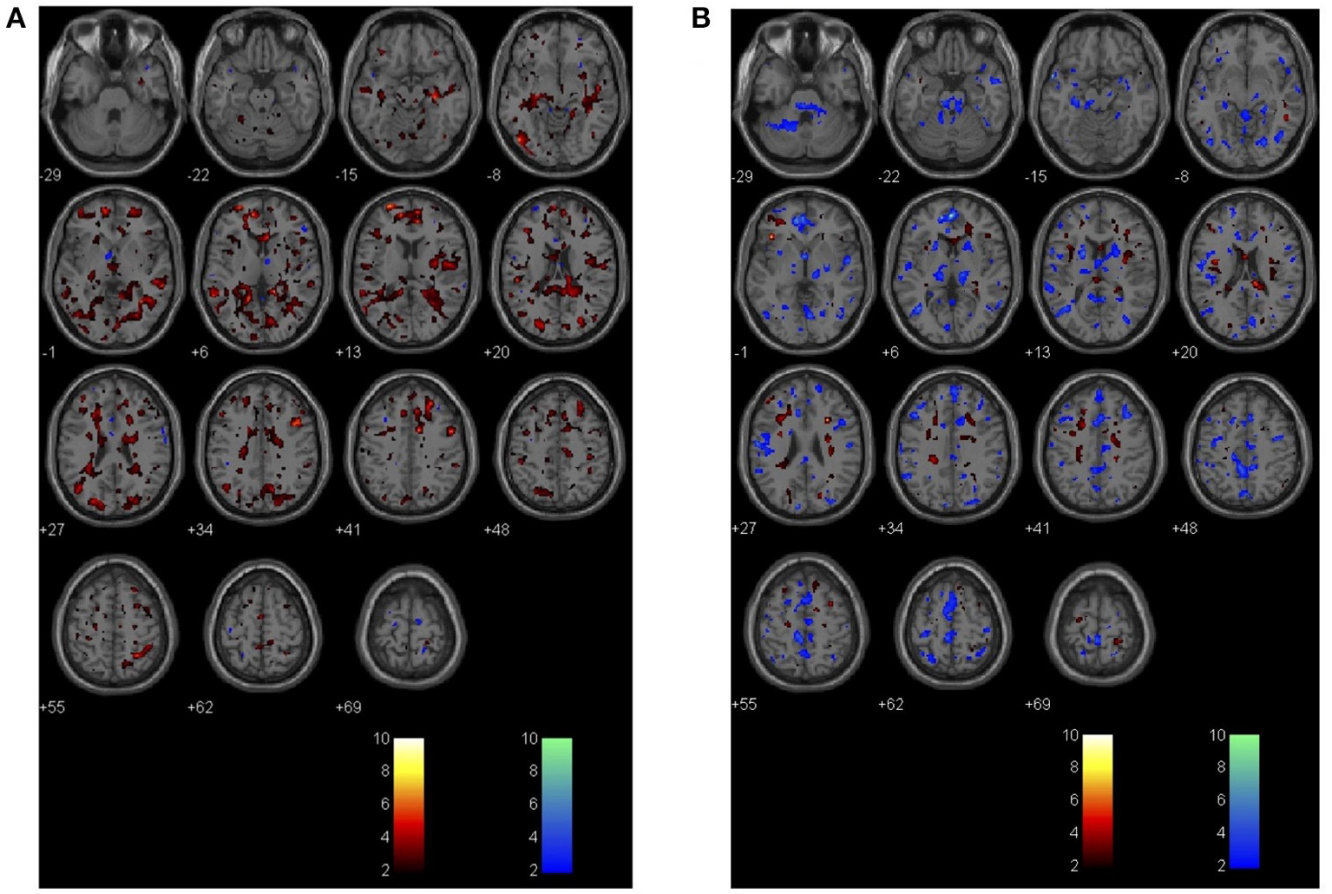
**(A)** Demonstrates 2nd Level Analysis in SPM comparing the non-POD group (*n* = 6) and the subthreshold (ST) group (*N* = 3). The hot voxels represent where the BOLD response to CO_2_ is significantly more pronounced in the non-POD or control group. The cold voxels indicate where the inverse BOLD response is significantly more in the ST group. The color bar is for *p* = 0.05 in this circumstance. **(B)** 2nd Level Analysis in SPM comparing the non-POD group (*n* = 6) and the POD group (*N* = 3). The hot voxels represent where the BOLD response to CO_2_ is significantly more pronounced in the non-POD or control group. The cold voxels indicate where the inverse BOLD response is significantly more in the POD group. The color bar is for *p* = 0.05 in this circumstance.

Figure 2A demonstrates 2nd Level Analysis in SPM comparing the non-POD group and ST-POD group. The hot voxels represent where the BOLD response to CO_2_ is significantly more pronounced in the non-POD group. The cold voxels indicate where the BOLD response is significantly less in ST-POD. Figure 2B demonstrates 2nd Level Analysis in SPM comparing non-POD group and the POD group. The hot voxels represent where the BOLD response to CO_2_ is significantly more pronounced in the non-POD or control group. The cold voxels indicate where the BOLD response is significantly less in the POD group. The color bar indicates significant *t*-values at the *p* = 0.05 level in both circumstances.

### Axial FLAIR imaging

**Figure 3:**
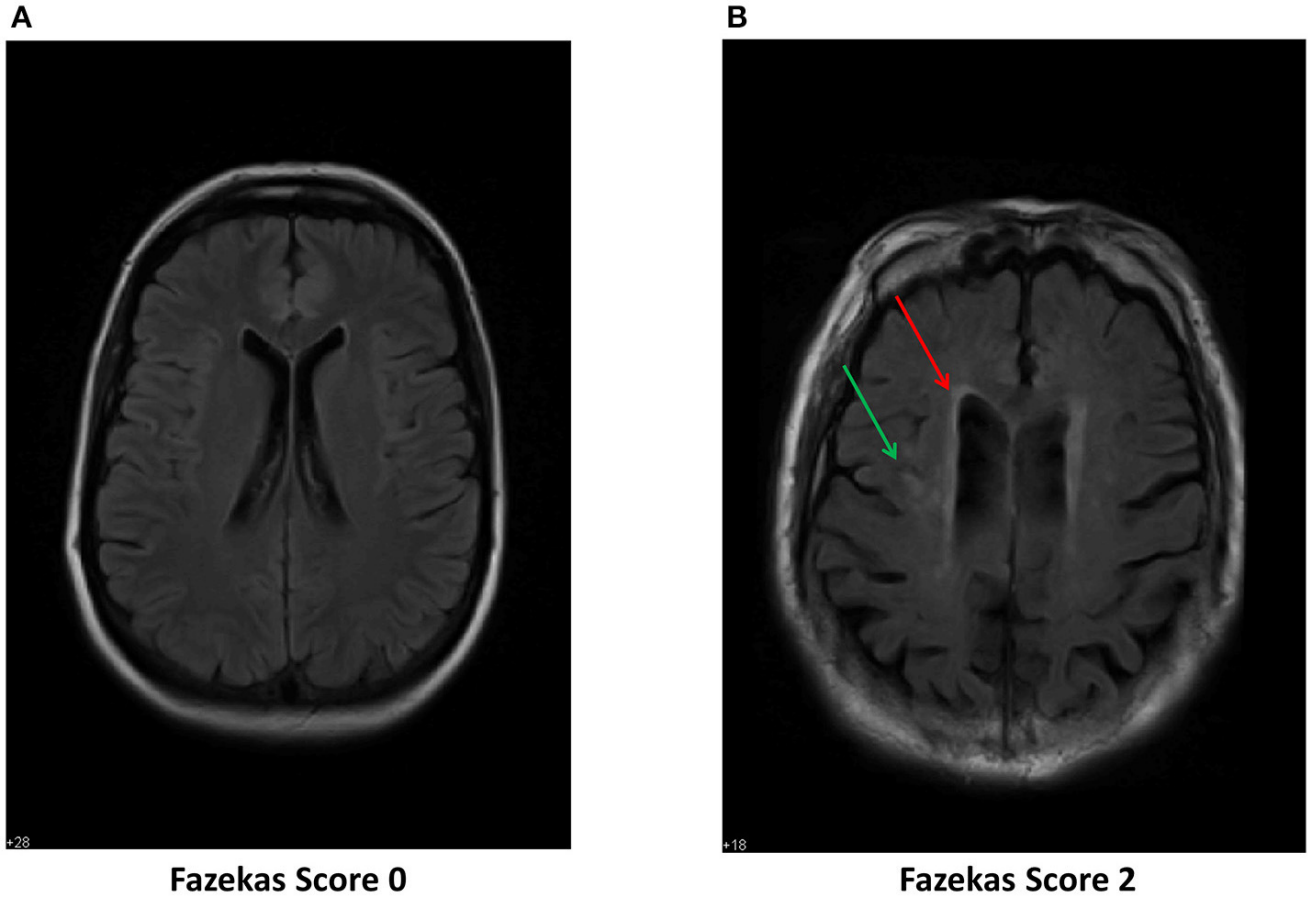
Axial FLAIR images of two of the study patients. **(A)** Shows normal white matter imaging. This patient was scored Fazekas Grade 0. **(B)** Shows areas of hyperintensity in the periventricular regions (red arrow) and in the deep white matter (green arrow). This signature has been identified as a marker for leucoaraiosis. This image was blindly scored and on neuro-radiology report identified as Fazekas Grade 2.

Axial FLAIR images were examined for evidence of leucoaraiosis—a white matter lesion associated with BOLD image CVR changes and later life dementia (Sam et al., 2016a,b). Figure 3A demonstrates an axial FLAIR image of one of the non-POD patients and Figure 3B a POD patient. The Figure 3B shows areas of hyperintensity in the periventricular regions and in the deep white matter.

### Neuropsychological and psychiatric factors

Table 1 displays percentiles of each significant neuropsychological factor, and the presence or absence of psychiatric illness across all participants. Not shown, bivariate correlations revealed that certain neuropsychological measures, including Animal Fluency scaled score (*r* = −0.90, *p* < 0.001), DKEFS Color Word Interference Condition 1 (*r* = −0.76, *p* = 0.006) and Condition 2 (*r* = −0.73, *p* = 0.01) scaled scores, and CLOX 1 z-score (*r* = −0.83, *p* = 0.02) were significantly correlated with peak POD score, such that worse scores on these measures pre-operatively were associated with higher post-operative peak POD scores. Bivariate correlations also indicate Animals (*r* = −0.81, *p* = 0.002) and DKEFS Color Word Interference Condition 1 (*r* = −0.76, *p* = 0.007) and 2 (*r* = −0.70, *p* = 0.002) were significantly associated with average POD severity score. Linear regressions corroborated these findings. Multiple linear regressions controlling for age, education, sex, and baseline CAM-S revealed that Animal Fluency (β = −1.2, *p* < 0.001), DKEFS Condition 1 (β = −1.6, *p* = 0.003) and Condition 2 (β = −1.0, *p* = 0.01) were significantly associated with peak POD score, but CLOX 1 was non-significant. Results were corroborated for multiple linear regressions with average delirium score as the dependent variable.

With respect to psychiatric factors, results revealed that PHQ-9 summary score was bivariately correlated to average delirium severity score (*r* = 0.58, *p* = 0.049). Adjusted linear regressions were non-significant.

### Intra-operative stress

Intra-operative stress is summarized in Table 2 for each participant. Contributory trends emerged for the following stressors—mean blood pressure less than 60 mmHg, end-tidal CO_2_ delta greater than 10 mmHg, cerebral saturation less than 60% and surgical duration greater than 120 min. Anesthetic stress defined by these factors descriptively appear to contribute to POD outcomes, acting as either a catalyst in the case of few diathesis risk factors and high intra-operative stress, or a protective factor in the case of diathesis risk and low intra-operative stress. There were no significant differences in post-operative narcotic dosing intra-operatively or post-operatively between the 3 groups (shown in Table 3).

**Table 3:** Distribution of demographic, diathesis, stressor, and delirium severity indicators across delirium groups.

	**No delirium (*n* = 6)**	**Subthreshold delirium (*n* = 3)**	**Full delirium (*n* = 3)**	**F Statistic**
**DEMOGRAPHICS**				
Age	63.5 (12.2)	64.3 (9.5)	56 (9.6)	0.6
Education	12.5 (2.3)	13.3 (2.1)	13.3 (2.3)	0.2
% female	16.7%	66.7%	66.7%	NS
**POTENTIAL DIATHESIS FACTORS (EXCLUDING VOXEL COUNT)**				
Pre-operative CO_2_ delta	4.8 (0.9)	4.6 (1.1)	5.1 (1.0)	0.2
Depressive symptoms (PHQ-9 Sum)	0.8 (1.0)	5.3 (4.0)	7.0 (4.6)	5.1^*^
Anxiety symptoms (GAD-7 Sum)	0.7 (0.5)	7.0 (8.7)	9.7 (7.0)	3.4
% illicit drug use past 2 weeks	0%	0%	33.3%	NS
**NEUROPSYCHOLOGICAL FACTORS (PERCENTILES)**				
Semantic fluency (Animals)	71.3	62.3	15.7	9.4^**^
Processing/executive functioning (DKEFS color word interference condition 1)	50.2	54.0	9.0	6.1^*^
Processing/executive functioning (DKEFS color word interference condition 2)	55.0	43.7	31.7	1.1
Spatial skills (CLOX 1)	82.8	47.0	56.5	18.9^**^
**POTENTIAL STRESSOR FACTORS**				
Length of Surgery	216.0 (123.8)	228.0 (192.3)	225.3 (138.1)	0.0
Intra-operative CO_2_ Delta	12.1 (3.9)	14.5 (5.2)	12.3 (2.8)	0.4
Cerebral SAT (0 or 1)	0%	0%	66.7%^*^	*p* = 0.03^*^
Mean MAC	0.95 (0.13)	0.90 (0.0)	0.77 (0.29)	1.0
Intra-operative morphine equivalence (mg)	24.6 (17.3)	17.3 (11.2)	17.0 (7.2)	0.4
Total post-operative morphine equivalence (mg)	43.0 (28.0)	100.6 (99.1)	135.2 (78.0)	2.4
**DELIRIUM FACTORS**				
Average severity	1.9 (1.4)	3.5 (1.1)	6.9 (2.9)	7.9^**^
Peak post-operative severity	2.5 (1.1)	5.5 (0.5)	11.9 (3.4)	27.7^***^
Days in Hospital	3.0 (1.8)	4.7 (1.5)	5.7 (0.6)	3.3

* p < 0.05,

** p < 0.01,

*** *p < 0.001. Analyses of variance were conducted for continuous variable and chi-square for categorical variables where percentages are reported and significance if applicable. For neuropsychological factors, F statistic and p-value based on scaled score for DKEFS condition 1 and 2, and Standard Score for Animals and CLOX 1. Percentiles reported for interpretability. NS, non-significant; PHQ-9, Patient Health Questionnaire – 9 item; GAD-7, Generalized Anxiety Disorder Scale – 7 item; DKEFS, Delis-Kaplan Executive Functioning System*.

### Stress-diathesis summary findings

Table 3 demonstrates mean scores of primary variables associated with POD across the 3 POD groups. Significant differences in peak POD scores and average POD scores were demonstrated for non-POD, ST-POD, and full POD groups. Corroborating previous regression findings, results also indicated significant differences across POD groups for depressive symptoms, and percentile scores (displayed for interpretability) on semantic fluency and processing speed/executive functioning on neuropsychological testing. A chi-square analysis indicated a significant difference for cerebral saturation (SAT), indicating only those with full POD had cerebral SAT less than 60%.

**Figure 4:**
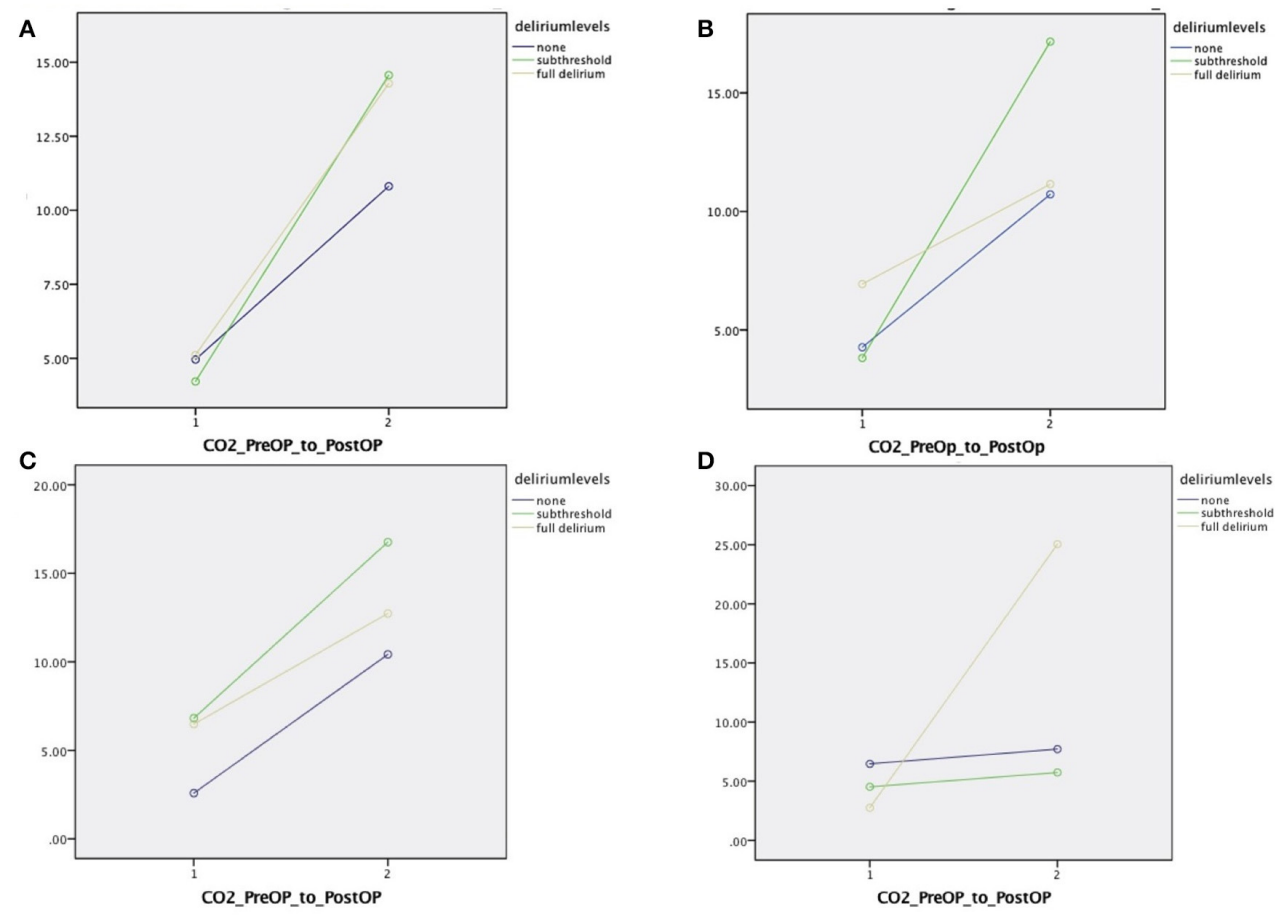
**(A)** Repeated measures mixed effect analysis of variance examining change score in CO_2_ delta pre- and post-measurement across POD levels adjusting for sex, education, and age. **(B)** Controls for semantic fluency scaled score, processing speed scaled score (DKEFS color word interference condition 1 and 2 scaled score mean), and history of psychiatric illness. **(C)** Controls for sociodemographics and Type 1 and 2 hypercapnic responses from white and gray matter. **(D)** Controls for sociodemographics and duration of surgery, cerebral SAT < 60, and mean MAC.

**Figure 5:**
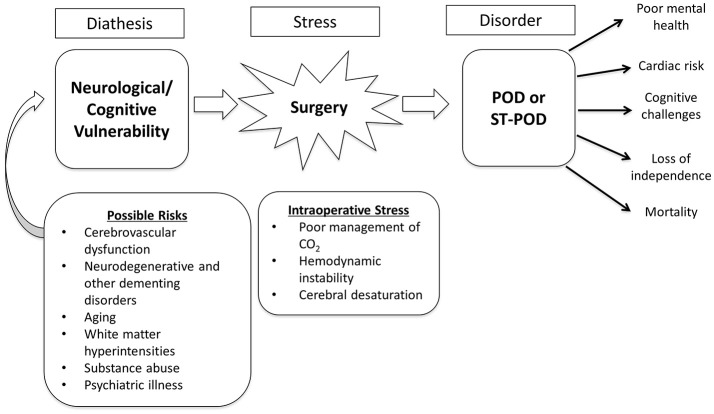
Diagrammatic depiction of the stress-diathesis model for POD and ST-POD. See text for further details.

Figures 4A-D demonstrates graphical results from the repeated measure analysis of covariance. The Y-axis in all graphs represents marginal means, and the X-axis represents pre-operative CO_2_ delta as indicated by the stress test, and intra-operative CO_2_ delta. Figure 4A includes age, sex, and education as covariates in the model, demonstrating larger intra-operative CO_2_ delta for ST-POD and POD compared to non-POD. Figure 4B controls for sociodemographics, significant neuropsychological factors, and presence of a psychiatric disorder. As demonstrated, intra-operative stress is attenuated for the POD group, while maintaining significant diathesis CO_2_ elevation pre-operatively. Intra-operative stress of CO_2_ delta is significantly higher for ST-POD, than non-POD and POD groups. Figure 4C controls for sociodemographics, and pre-operative voxel count for gray and white matter in hypercapnic response and inverse hypercapnic responses. Controlling for this diathesis results in pre-operative elevation in CO_2_ delta for ST-POD and POD groups relative to non-POD, and a risk gradient for intra-operative CO_2_ delta, where ST-POD demonstrated the largest marginal mean, followed by POD, and non-POD. Figure 4D controls for socio-demographics and intra-operative stressors, results dramatically change where POD has higher intra-operative CO_2_ delta marginal means compared to non-POD and ST-POD groups. Figure 5 displays the proposed stress-diathesis model of POD and ST-POD incorporating these factors.

### Post-operative delirium follow-up

All 12 patients were successfully reached at follow-up (time since surgery range = 1 to 6 months). In the non-POD group, 5 participants reported no change in thinking, and one discussed occasional age-related lapses in short-term memory that was not noticed by loved ones. In the ST-POD group, 1 participant reported no change in cognition, and two indicated marked changes in cognition since surgery, providing examples such as “when picking up groceries I would forget what store I was in,” “I tell the same story over and over.” Of the full POD group, two participants described significant changes in memory and one did not. One POD patient reported that their spouse noticed significant cognitive changes and provided examples such as the more minor incident of “forgetting appointments,” to the more extreme of “forgetting I am taking care of a newborn.” The other POD patient was nonsensical, and had significant difficulty responding to any direct questions. This participant was able to indicate that they did not recall being home post-discharge for the first number of days. This person was assessed 4.5 months post-surgery.

## Discussion

The spectrum of acute confusional states following surgery, most commonly referred to as POD on the severe end of the spectrum, remains a perplexing and serious problem. Its management and consequences are time consuming, prolong hospital stay and have serious effects on long-term patient well-being (Vasunilashorn et al., 2016). A means to accurately predict those patients at risk would hopefully guide intra-operative and post-operative management and permit formal studies to more accurately address the problem. To the best of our knowledge, this study represents the most comprehensive, multidisciplinary approach to identify biomarkers of POD to date. We have assessed patients with a detailed neuropsychological battery prior to surgery, evaluated pre-operative psychiatric history, documented risk of intra-operative steal of cerebral blood flow for brain regions at risk by advanced neuroimaging techniques (MRI BOLD and axial FLAIR imaging) and tracked hemodynamics, end-tidal gases and cerebral oximetry in high fidelity intra-operatively to look for risk factors of POD. We also include a comprehensive POD assessment method, which derives severity scores and the ability to identify subthreshold manifestations, and longitudinally followed up with all participants.

We report on 12 adult patients receiving major surgery at a single site. As such this report represents a pilot study to inform the development of a stress-diathesis metric delineating risk of POD. We suggest that identifying psychiatric, neuropsychological (cognitive), and neurophysiological risk factors using symptom measures along with a pre-operative brain MRI CO_2_ stress test and other advanced neuroimaging approaches, combined with assessment of intra-operative events, namely deviations in the conduct of anesthesia, can be predictive of ST-POD and POD. Based on the observations from this study, we also advance the idea that adherence to a brain protective protocol whereby normocapnia is maintained (or more precisely prevention of significant alterations in intra-operative CO_2_ delta) may help decrease the incidence of POD and ST-POD in this patient population.

### Diathesis findings

The MRI BOLD CO_2_ stress test (i.e., pre-operative exposure to graded CO_2_ while undergoing neuroimaging) was employed as a separate indicator of risk in our stress-diathesis model to be used as a proxy for intra-operative stress. This procedure specifically involves a fixed vasodilatory stimulus that was administered to all patients—a CO_2_ delta targeted to 5 mmHg during normoxic isoxia. This test has been successfully used to assess risk in other clinical scenarios. Risk stratification has been identified in patients with severe cerebrovascular compromise such as moya-moya disease, atherosclerotic stenosis or occlusion and more recently for concussion, white matter changes (leucoaraiosis) and dementia (Mandell et al., 2008; Fierstra et al., 2011; Han et al., 2011; Gao et al., 2013; Mutch et al., 2014, 2015; Sam et al., 2016a,b). All of these conditions have demonstrations of altered cerebrovascular reactivity, which become unmasked by controlled administration of CO_2_ as a potent vasodilatory stimulus. In the present study, differences in response to CO_2_ are evident between the patients without POD vs. those that manifested either full or ST-POD, with the most robust findings for full POD (see Figures 1, 2). What is evident from the small case series here is that POD patients have a decreased response to the hypercapnic signal in gray and white matter (Figure 1B) and a trend to greater inverse response or intracranial steal (Figures 1B, 2B). These findings have also been identified with a group of patients demonstrating leucoaraiosis (Pantoni, 2008; Figure 3). Quantification of these risks and their distribution requires an atlas of age matched surgical patients without POD to determine voxel thresholds for risk of POD. In this manner voxel count “cut points” can be determined. What is further evident from examination of these images is that there are various subgroups that help to define risk of POD. There is a group with essentially normal response to hypercapnia—both in gray and white matter—who did not demonstrate POD irrespective of the anesthetic course. Based on this finding there appears to be an identifiable low risk group defined by the CO_2_ stress test. Another subgroup had an attenuated response to the hypercapnic stimulus and a greater signature for intracranial steal (both markers of cerebral dysregulation). This group is identified as high risk for POD. Whether or not these individuals manifested POD appeared to depend on both other pre-operative risk factors (e.g., neuropsychological functioning and psychiatric history) and their management during the anesthetic course (see Tables 1, 2, and further discussion below). A third subgroup had smaller signatures for CO_2_ responsiveness and had a mixed response as to POD presentation. Importantly, in all patients the CO_2_ delta was greater than a CO_2_ delta targeted to 5 mmHg (during the CO_2_ stress test) during the intra-operative course as shown in Supplemental Table 1. This suggests that our CO_2_ stress test was very conservative to unmask risks of intracranial steal. Despite this, this change in CO_2_ delta from the pre-operative stress test to the intra-operative period helped consolidate the stress-diathesis framework. As evident in Figure 4, including diathesis-related covariates (i.e., a) implicated neuropsychological vulnerabilities and presence of a clinically significant psychiatric profile, and b) intracranial steal responses during CO_2_ stress test) greatly affect the full POD group, while including intra-operative stress covariates (i.e., duration of surgery, cerebral SAT < 60, and mean MAC) greatly affect the ST-POD group. This may suggest that intra-operative stress may play a larger role in ST-POD patients, a group with less pre-operative diathesis risk. With a larger sample size, rigorous prediction models will allow for a greater understanding of the diathesis and stressor contributions in POD.

With respect to pre-operative neuropsychological risk factors, preliminary data revealed that cognitive measures of semantic categorical fluency and information processing and speed/executive functioning were strongly associated with POD severity. This may correspond with vulnerabilities in temporal and frontal regions of the brain. Interestingly, POD severity was unrelated to measures of pre-operative phonemic fluency (F-A-S) despite the strong effect on semantic fluency; this discrepancy has been well documented as an indicator of Alzheimer's disease (Henry et al., 2004), which may represent an underlying contributor to POD. Relatedly, prior research has found that delirium accelerates cognitive decline in Alzheimer's disease, which may suggest related pathophysiology (Fong et al., 2009). The findings specific to these measures, which may reflect particular regional deficiencies, emphasizes the importance of examining a wide range of measures assessing specific cognitive abilities. Establishing an atlas of POD-free subjects could aid in determining if the MRI BOLD CO_2_ stress test can be declarative in modeling these neuropsychological deficits based on attenuated response to the hypercapnic stimulus or an intracranial steal signature. This is important in order to use neuropsychological measures as a proxy for at-risk patients, as the administration of a pre-operative MRI BOLD CO_2_ stress test is not feasible in general practice. The MMSE, which is the most widely used assessment tool in POD research, did not differentiate POD groups, nor was it associated with POD severity. Finally, as indicated in prior research, psychiatric illness emerged as being associated with POD, namely depressive symptoms. The emerging diathesis profile is similar to deficits reported in white matter hyperintensities and subcortical vascular dementia such as leukoaraiosis, characterized by depressive symptoms, motor and gait disturbances and cognitive deficits. In further support, in healthy older adults, white matter integrity is correlated with greater processing speed (Kerchner et al., 2012), which may suggest that the deficiencies in processing speed measures (DKEFS) is associated with reduced white matter integrity, which was correlated with POD.

### Stressor findings

The stressor was defined as the intra-operative anesthetic course. High fidelity hemodynamic and end-tidal gas output and continuous bifrontal cerebral saturation by cerebral oximetry were obtained using an intra-operative data acquisition system that downloaded the data stream at 0.5 hz (TrendFace Solo). These data were then binned to a minute-by-minute frequency. The data that were included to define the intra-operative stress and binarized were hypotension (mean blood pressure < 60 mmHg for a minimum of 1 min), elevated CO_2_ delta > 10 mmHg, duration of surgery greater than 120 min, and cerebral saturation <60% for greater than 1 min).

Although preliminary, our data demonstrate that inducing stress via CO_2_ exposure pre-operatively allows for an index of brain response intra-operatively. Those demonstrating diathesis risk through specific cognitive vulnerabilities described above, psychiatric illness, and abnormal response to CO_2_ stress are at highest risk of POD. As previously indicated, although several diathesis risk factors are linearly related to the severity of POD, it is hypothesized that the intra-operative anesthetic course will not demonstrate a clear linear relationship with outcomes, and may be more interactive.

The development of this stress-diathesis hypothesis, exhibited in Figure 5, is based on the current findings in combination with a number of recent findings that both emphasize the multifactorial nature of POD, the null findings with respect to anesthetic toxicity, studies demonstrating pre-existing structural and functional abnormalities in those with POD (Soiza et al., 2008), and the recent findings demonstrating the importance of pre-operative cognitive dysfunction in POD. One recent study examining pre-operative arterial spin labeling MRI using whole brain and globally normalized voxel wide analysis found that greater performance on neuropsychological measures correlated with cerebral blood flow, but these neuroimaging findings were not predictive of the risk of POD (Hshieh et al., 2016). Importantly, however, their study did not incorporate an assessment of intra-operative stress-related factors that would impact brain pathophysiology, which in isolation limits our understanding, as we believe it is the interaction between these brain vulnerabilities and the stress response (the stress-diathesis) that is predictive of both POD and ST-POD. Future research should aim to examine the relationship between cognitive deficiencies, and CO_2_ stress test using BOLD MRI in order to understand whether these aforementioned correlations extend to emergent intracranial steal that may be anatomically indicated in part by leucoaraiosis or associated with alterations in cerebrovascular responsiveness in dementias—specifically as noted with Alzheimer's Disease.

Our findings are revealing and provocative. We acknowledge the small sample size of our study as a primary limitation. Despite this, we have identified biomarkers in neuropsychological testing and the psychiatric realm and MRI BOLD CO_2_ stress test that when combined together may be predictive of POD. In addition, our sample size (*n* = 12) has been deemed large enough to adequately power fMRI studies for lower percent changes in activation of BOLD signal (Desmond and Glover, 2002). We have provided preliminary support for a stress-diathesis model based on these interactions and observations. Another limitation relates to defining features of subthreshold and full POD. We chose to use a newly validated POD severity measure to establish severity scores but acknowledge this may not fully encompass other potential markers such as the full spectrum of post-operative confusional states or low-grade encephalopathic states. However, our long-term follow-up provided clinical validity to these severity groups. Specifically, two-thirds of ST-POD or POD patients reported significant changes in cognition since surgery, with one who may have continued to meet criteria for POD over 4 months post-operatively. In comparison, 5/6 non-POD participants reported no change in memory following surgery, with one indicating potential mild age-related changes, which had not been corroborated by a loved one. Nonetheless, there are a number of etiological mechanisms that may manifest differently in acute confusional states in other contexts that warrants further exploration.

### Clinical implications

As previously indicated, neuroprotection of POD through anesthetic agents has not been supported. However, other means are available to provide neuroprotection to patients undergoing anesthesia for surgery in the operating room. Neuroanesthesia principles are time-honored and designed to optimize the surgical approach to the brain. It would seem to make sense to apply such approaches to the management of patients at risk for POD. While mild hypocapnia is usually maintained in neurosurgical procedures to reduce brain bulk, our findings here suggest that maintenance of normocapnia minimizing CO_2_ delta intra-operatively may be the best course to choose for patients at risk of POD. There are older animal studies to support this contention. In two studies done using a paraplegia model, spinal cord was examined as a focus of injury. In these studies clear end-points of damage and comprehensive hemodynamics and end-tidal gases were reported. The first paper shows no difference between intravenous anesthesia (methohexital) or volatile agent (isoflurane) on the incidence of paraplegia suggesting the anesthetic agent is not at issue (Mutch et al., 1993b). The second paper shows a clear advantage of a neuroanesthesia approach (Mutch et al., 1993a).

An anesthetic approach tailored to rigorous control of intra-operative CO_2_ may be appropriate as we now have greater understanding of the impact of alterations on CBF with changing levels of CO_2._ When the brain manifests with cerebrovascular dysregulation, conditions exist for intracranial steal with elevation of CO_2_ and the possibility of augmented flow to these areas with mild hypocapnia—the so-called “Robin Hood” effect. However, this effect is lost with subsequent elevation of the CO_2_ tension leading to steal with return to normocapnia. This study has revealed the magnitude of change in CO_2_ that can occur with major surgery. The mean delta was 13.2 ± 2.9 mmHg; range 7.5 – 20.0 mmHg. Thus, none of the patients had a CO_2_ delta as low as the hypercapnic stimulus in the brain MRI CO_2_ stress test. For this reason the voxel counts reported are conservative estimates of the response to CO_2_ in any given patient. The effect of CO_2_ intraoperatvely could be further confounding as all patients received volatile agents—known cerebrovascular vasodilators. Patients are also at ischemic risk with hypotension and in this context the combination of hypotension with larger CO_2_ deltas would be anticipated to be even more deleterious. An operative procedure where such a situation can routinely arise is with open aortic aneurysmectomy. With cross-clamp release hypotension in combination with large swings in CO_2_ ensues. It has been noted that this patient group suffers with a very high incidence of POD (Salata et al., 2012). Especially important in the context of the MRI BOLD CO_2_ stress test is delineating the magnitude of intracranial steal in brain at risk. Follow up studies where the brain stress test utilizes a greater CO_2_ delta are being entertained; most likely using a ramp protocol of incremental CO_2_ change that is better tolerated by patients, but able to examine a greater range (Fierstra et al., 2013; Sobczyk et al., 2014; Supplemental Video 1).

Importantly, maintenance of normocapnia as a prevention effort may also extend to intensive care settings, where POD rates are also high. Many of these patients will have the same diatheses, even the same stress if managed in a surgical ICU, following their operative intervention. Irrespective of management in a medical or surgical ICU, mechanical ventilation is extremely common, with swings in end-tidal CO_2_ evident, often with hemodynamic instability. In fact, in intensive care units POD has been found to be associated with longer durations of mechanical ventilation (van den Boogaard et al., 2012), supporting this contention.

## Conclusion

This study calls into question current conceptualizations of ST-POD and POD, significant and deleterious neuropsychiatric syndromes. Focus, to date, has been on modifying or limiting exposure to anesthetic agents. Our study suggests that an uncontrolled stress-diathesis may be driving high rates of ST-POD and POD. Specifically, preliminary data suggest that diatheses may be present that put particular patients at risk, particularly depressive symptoms, impaired semantic fluency and processing speed/executive functioning, and pathophysiological vulnerabilities that are evident on neuroimaging during exposure to controlled CO_2_ prior to surgery. The latter represents a proxy for the proposed intra-operative stressor—fluctuations in end-tidal CO_2_ during surgery, which may act as a catalyst for the expression of the spectrum of acute confusional states. We propose that the stressor itself could be mitigated by tight control of CO_2_ in the normocapnic range acting as “neuroprotection” for those deemed at risk. A version of the stress-diathesis risk assessment as described here with comprehensive neuropsychological testing provides the testing platform for future investigations (Enzinger et al., 2007).

## Ethics statement

This study was carried out in accordance with the recommendations of the Biomedical Research Ethics Board (BREB) of the University of Manitoba with written informed consent from all subjects. All subjects gave written informed consent in accordance with the Declaration of Helsinki. The protocol was approved by the Biomedical Research Ethics Board (BREB) of the University of Manitoba.

## Author contributions

RE and WM contributed equally to this work including idea development, study design, methodology, personnel management, statistical analyses, and co-wrote the primary draft of the manuscript. RP contributed to design, methodology, personnel management, and reviewed drafts of the manuscript. KK contributed to methodology, participant recruitment, assessment, data collection and entry, and reviewed drafts of the manuscript. CB contributed to methodology, participant recruitment, assessment, data collection and entry, and reviewed drafts of the manuscript. CH contributed to methodology, participant recruitment, assessment, data collection and entry. LR contributed to MRI data collection, SPM processing and analysis. DF contributed to data collection and interpretation, and coordinated intra-operative management. RL contributed to data collection, entry, and management. JF, JD and DM contributed to idea development, study design, methodology, and interpretation of findings.

### Conflict of interest statement

JF, JD, and DM have patent rights on the RespirAct™ device described in this manuscript. They and the University of Toronto stand to gain financially if the device described is sold commercially. The other authors declare that the research was conducted in the absence of any commercial or financial relationships that could be construed as a potential conflict of interest.

## Supplementary material

Supplemental Figure 1 Explanation for intracranial steal demonstrated with the BOLD MRI CO_2_ stress test as described in this study. **(A)** The CO_2_ stress administered is shown at the bottom of the panel. This was an 11-minute stimulus (660 s). A block design triple hypercapnic stimulus each of 2 min at 5 mm Hg increase in CO_2_ was interspersed with baseline end-tidal CO_2_. Importantly the end-tidal O_2_ was “clamped” at approximately 110 mmHg during the changes in CO_2_ (this response is not shown). In the middle row the BOLD response at one voxel is shown for the anticipated response BOLD signal increase with hypercapnia—an increase in cerebral blood flow (CBF) compared with baseline CO_2_ is shown for the triple stimulus (highlighted in gray). The red dots depict a single scan BOLD signal response (330 dots or one scan every 2 s). The tight response to the triple stimulus is shown in the left panel. The inverse response in one voxel is shown in the right panel –greater BOLD signal at baseline CO_2_ and BOLD signal decreasing with hypercapnia—a paradoxical response as CBF has decreased with increasing CO_2_—an example of intracranial steal in this voxel. The top panel shows the distribution of voxels for the two responses (so-called “glass brain” depiction). A voxel is gray—increasing hue as the *t*-value statistic increases above the cut point at the chose *p*-value (*p* = 0.001 in this circumstance). Most voxels demonstrate the expected behavior to the stimulus. The right top panel shows those voxels—far fewer in number - that demonstrate the inverse response or intracranial steal. **(B)** The glass brain statistical distribution of voxels is now colorized in the left panel. Increasingly hot colored (orange hues) voxels show where CBF has increased in response to the CO_2_ stimulus. The cold-colored (blue hue) voxels show regions of intracranial steal. These are largely in periventricular or deep white matter regions (fuller explanation in the text). A parallel flow model showing contiguous regions explaining intracranial steal and normal response is shown in the right panels. With flow-limited input (the CBF response is flow limited and demonstrates a sigmoid response to increasing CO_2_) normally flow increases regionally with increasing CO_2_ and the BOLD signal increases – shown as increasing orange hues in the micro-regional (green circle) distribution. In diseased areas with abnormal response (vasoparalysis) flow can be stolen from this region as the vasculature is already maximally vasodilated and thus paradoxically “feeds” the normal vasodilating adjacent region. This is depicted as increasing bluish hue in the green vascular circle. The green circle shows such a region in the colorized image with a blue steal region next to a normal orange region. The parallel path for the two regions is shown to the right with normal vasomotion shown as an arrow directed outward from the feeding vessel and abnormal vasomotion shown as a double headed arrow with paralyzed effectiveness. The normal response is highlighted by the green circle in the lower figure (showing hot coloration throughout and increasing orange hue depicting increased CBF). The parallel distribution is shown in the bottom right panel. Normal vasomotion occurs in both parallel arms in the depiction to the right of this region.

Supplemental Video 1 This video depicts the dynamic response of the human brain to alterations in end-tidal CO_2_ tension. The left panel shows the CO_2_ stimulus over time. The CO_2_ delta here is approximately 20 mmHg—the upper limit of change in CO_2_ seen in this study. The right panel shows the changes in BOLD CVR to the CO_2_ stimulus. The signal changes to an essentially cold response (blue voxels) indicating decreased BOLD response with hypocapnia (an index of decrease cerebral blood flow (CBF) with a decrease in CO_2_). As the CO_2_ increases, increasing above the baseline value the brain response gets hotter (shades of orange). This correlates with an increase in CBF with the increase in CO_2_. A return toward baseline values occurs as the CO_2_ stimulus abruptly returns to the starting value. This ramp sequence indicates a more reflective CO_2_ delta for the intra-operative stress observed.

Supplemental Table 1 The end-tidal gases for each subject are shownbaseline CO_2_ (CO_2_ BL), CO_2_ delta, and mean O_2_ in mm Hg. A comparison between the non-POD and combined ST-POD- and POD-values and *t*-statistics are also shown.

Supplemental Data Sheet 1 A glossary of anesthesia terms relating to gas exchange and intracranial steal.
