# Supplementary figures and images for: A Novel Stress-Diathesis Model to Predict Risk of Post-operative Delirium: Implications for Intra-operative Management
Source: Front Aging Neurosci. 2017 Aug 18;9:274. doi: 10.3389/fnagi.2017.00274 (PMC5563326; doi:10.3389/fnagi.2017.00274)

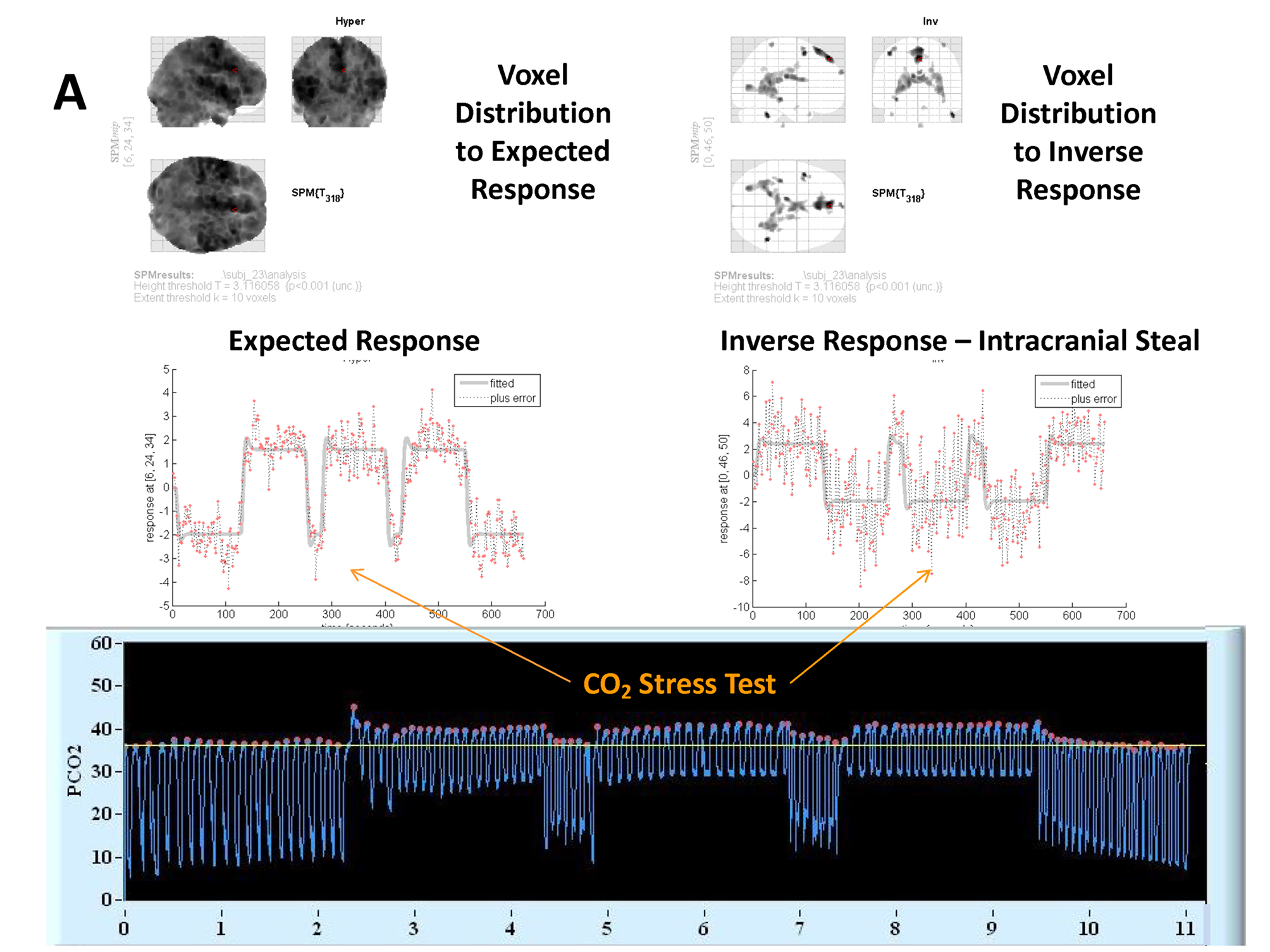

Supplement: Supplemental Figure 1 — Explanation for intracranial steal demonstrated with the BOLD MRI CO2 stress test as described in this study. (A) The CO2 stress administered is shown at the bottom of the panel. This was an 11-minute stimulus (660 s). A block design triple hypercapnic stimulus each of 2 min at 5 mm Hg increase in CO2 was interspersed with baseline end-tidal CO2. Importantly the end-tidal O2 was “clamped” at approximately 110 mmHg during the changes in CO2 (this response is not shown). In the middle row the BOLD response at one voxel is shown for the anticipated response BOLD signal increase with hypercapnia—an increase in cerebral blood flow (CBF) compared with baseline CO2 is shown for the triple stimulus (highlighted in gray). The red dots depict a single scan BOLD signal response (330 dots or one scan every 2 s). The tight response to the triple stimulus is shown in the left panel. The inverse response in one voxel is shown in the right panel –greater BOLD signal at baseline CO2 and BOLD signal decreasing with hypercapnia—a paradoxical response as CBF has decreased with increasing CO2—an example of intracranial steal in this voxel. The top panel shows the distribution of voxels for the two responses (so-called “glass brain” depiction). A voxel is gray—increasing hue as the t-value statistic increases above the cut point at the chose p-value (p = 0.001 in this circumstance). Most voxels demonstrate the expected behavior to the stimulus. The right top panel shows those voxels—far fewer in number - that demonstrate the inverse response or intracranial steal. (B) The glass brain statistical distribution of voxels is now colorized in the left panel. Increasingly hot colored (orange hues) voxels show where CBF has increased in response to the CO2 stimulus. The cold-colored (blue hue) voxels show regions of intracranial steal. These are largely in periventricular or deep white matter regions (fuller explanation in the text). A parallel flow model showing contiguous regions explaining i [file Image1.TIF]

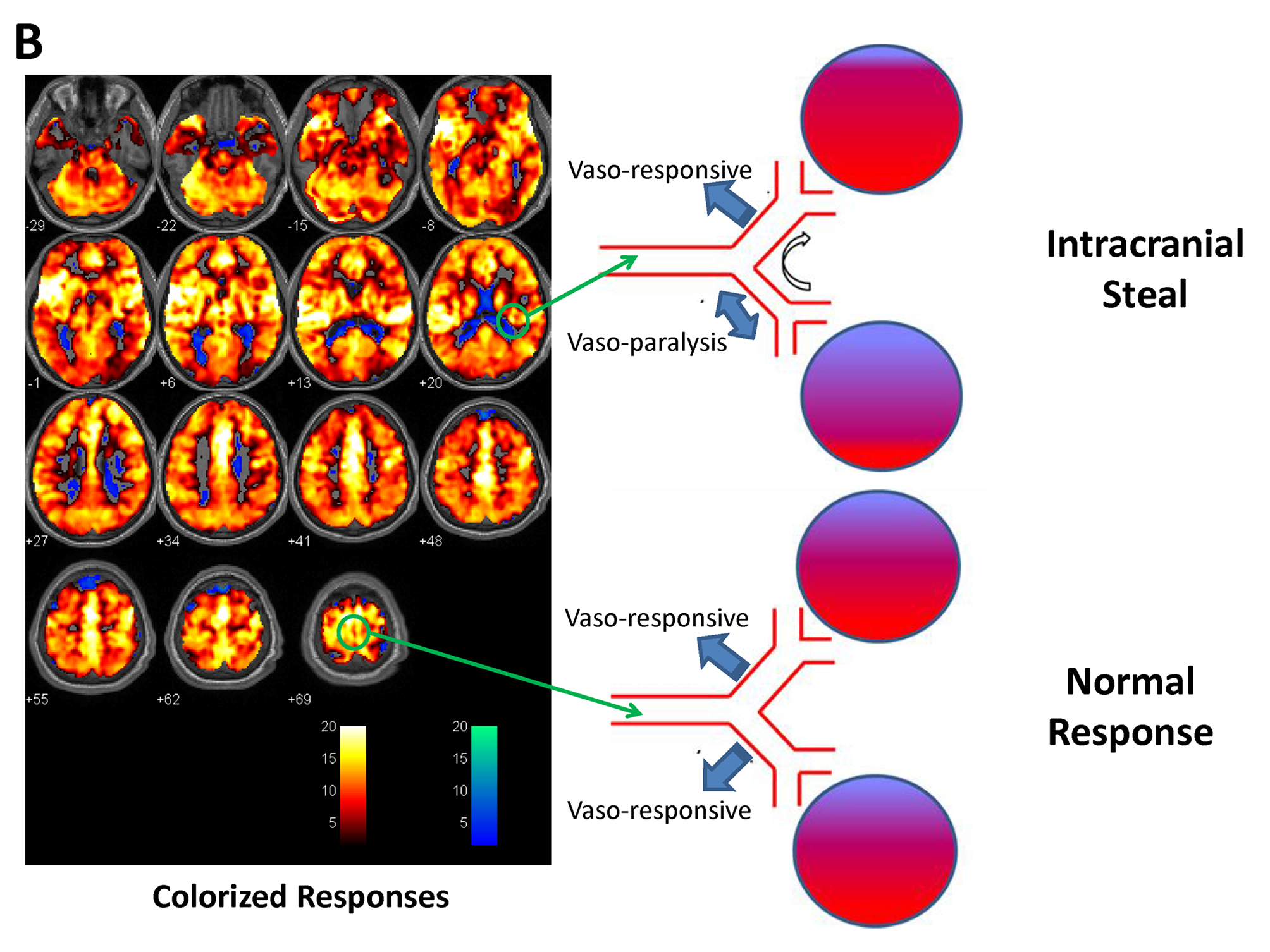

Supplement: Supplementary file 5 [file Image2.TIF]
