# Supplementary material for: A Novel Stress-Diathesis Model to Predict Risk of Post-operative Delirium: Implications for Intra-operative Management
Source: Front Aging Neurosci. 2017 Aug 18;9:274. doi: 10.3389/fnagi.2017.00274 (PMC5563326; doi:10.3389/fnagi.2017.00274)
Supplement: Supplemental Table 1 — The end-tidal gases for each subject are shownbaseline CO2 (CO2 BL), CO2 delta, and mean O2 in mm Hg. A comparison between the non-POD and combined ST-POD- and POD-values and t-statistics are also shown. [file Table1.docx]

| **End-Tidal Gases** | |  |  |
| --- | --- | --- | --- |
|  |  |  |  |
| Subject | CO_2_ BL | CO_2_ Delta | O_2_ Mean |
| 1 | 43 | 4.1 | 110 |
| 2 | 39 | 5.1 | 113 |
| 3 | 36 | 3.8 | 106 |
| 4 | 40 | 5.0 | 114 |
| 5 | 41 | 5.4 | 115 |
| 6 | 37 | 3.4 | 117 |
| 7 | 37 | 5.1 | 113 |
| 8 | 33 | 4.4 | 113 |
| 9 | 34 | 5.3 | 112 |
| 10 | 29 | 6.1 | 113 |
| 11 | 35 | 4.2 | 109 |
| 12 | 37 | 6.2 | 111 |
| **mean** | **37** | **4.8** | **112** |
| **SD** | **4** | **0.9** | **3** |
| **CV%** | **10** | **18.1** | **3** |
|  |  |  |  |
| **No-POD** |  |  |  |
| **mean** | 36 | 4.8 | 111 |
| **SD** | 2 | 0.9 | 3 |
|  |  |  |  |
| **POD** |  |  |  |
| **mean** | 38 | 4.9 | 114 |
| **SD** | 5 | 1.0 | 2 |
|  |  |  |  |
| **t-test** |  |  |  |
| **p-value** | 0.346 | 0.976 | 0.068 |
