# Supplementary material for: A Novel Stress-Diathesis Model to Predict Risk of Post-operative Delirium: Implications for Intra-operative Management
Source: Front Aging Neurosci. 2017 Aug 18;9:274. doi: 10.3389/fnagi.2017.00274 (PMC5563326; doi:10.3389/fnagi.2017.00274)
Supplement: Supplemental Data Sheet 1 — A glossary of anesthesia terms relating to gas exchange and intracranial steal. [file DataSheet1.PDF]

Glossary:

Normocapnia: normal levels of carbon dioxide (CO_2) gas tension in the arterial blood or at the end of expiration (end-tidal – as measured with a gas analyzer) with breathing – individualized but the mean range is usually 35 – 45 mmHg.

Hypocapnia: decreased levels of CO_2 tension in arterial blood or end-tidally. Commonly initiated by increasing minute ventilation under anesthesia during neurosurgical procedures. Occurs with tensions less than 35 mmHg in usual circumstances. Definitely present at tensions less than 30 mmHg. This condition is not necessary during other surgical procedures.

Hypercapnia: increased levels of CO_2 tension. Present with tensions above 45 mmHg in normal situations. Frequently present at the end of a surgical procedure as spontaneous ventilation is re-initiated while the patient is still under the influence of anesthetic agents and narcotics, which are respiratory depressants.

Minimum Alveolar Concentration (MAC): a measure of anesthetic depth for volatile anesthetic agents (administered by calibrated vaporizer with air:O_2 as inspired gas mixtures through an endotracheal breathing tube) based on the vapor concentration in volumes%.

Normoxic isoxia: Normal tensions of arterial or end-tidal oxygen (O_2) clamped at a constant tension (100-120 mmHg in this study).

Intracranial Steal: A pathological condition which can arise with redistribution of regional cerebral blood flow locally in the presence of upstream flow limitations causing competition for blood flow between downstream regions, particularly with vasodilatory stimulation; flow is preferentially distributed to those regions with more reactive vessels. This situation may occur with increases in CO_2 tension from decreased levels (hypocapnia) to normal levels (normocapnia) or from normocapnic levels to increased levels (hypercapnia). Volatile anesthetic agents can contribute to intracranial steal as they also cause cerebral vasodilation. **Supplementary Figure 1 A and B** diagrams how intracranial steal was determined for this study.

Brain BOLD MRI CO_2 Stress Test: An approach described in this study to determine brain-at-risk for post-operative delirium using a preoperative test. A standardized means to increase cerebral blood flow (CBF) is used (a tightly controlled increase in CO_2 tension) while measuring changes in blood oxygenation level dependent (BOLD) signal with MR imaging to assess the induced increase in regional CBF. The manner in which this is done is highlighted in **Supplementary Figure 1 A and B**. The brain-at-risk is associated with the regions identified with intracranial steal.
